# Supplementary material for: Perspectives of Women with Breast Cancer and Healthcare Providers Participating in an Adherence-Enhancing Program for Adjuvant Endocrine Therapy: A Qualitative Study
Source: Curr Oncol. 2025 Jan 17;32(1):45. doi: 10.3390/curroncol32010045 (PMC11764025; doi:10.3390/curroncol32010045)
Supplement: Supplementary file 1 [file curroncol-32-00045-s001.zip › Supplemantary Materiel S1.pdf]

Supplementary Material S1

INDIVIDUAL INTERVIEW GUIDE
PARTICIPATING WOMEN

OVERALL SATISFACTION WITH THE PROGRAM

- What do you think of the activities and tools you have received in the SOIE program?

- What did you like the most about the SOIE program? The less? For what reasons?

- To what extent the SOIE program's activities and tools have answered your needs?
    - To what needs it answered in particular?
    - For what reasons did the program answer or not your needs?
    - Did an activity or tool allow particularly answering your needs? For what reasons?

- Do you have the feeling that the SOIE program has made a difference on your experience with the anti-hormonal drug?
    - For what reasons?
    - What positive/negative impacts of the program have you perceived?
    - Did an activity or tool have a more significant impact than the others? If yes, which one and for what reasons?

- Did any factors facilitate/constrain your participation in the SOIE program?
    - What could have been done to facilitate your participation?

- Would you recommend the SOIE program to other women who have received an anti-hormonal drug after breast cancer? For what reasons?
    - Would you recommend an activity or tool in particular? If yes, which ones and for what reasons?

SATISFACTION WITH EACH PROGRAM' COMPONENT

For each program's component (educational group session/nurse-led telephone consultations/ chat sessions), the following questions were asked to the participant:

- What do you think of the time in your care trajectory when you have participated to the (COMPONENT)?
    - When would be the perfect time in the care trajectory?

- What do you think of the course of the (COMPONENT)?
    - What do you think of the content covered during the (COMPONENT)?
    - What do you think of the way the healthcare provider(s) delivered the (COMPONENT)?
    - *For the educational group session only:* What do you think of the means used to present the information? Of the delivery in a group format?
    - *For the chat sessions only*: What do you think of the platform used for the delivery of the chat sessions? What do you think of the sharing of experiences between the participants?

- What did you like the most about the (COMPONENT)? The less? For what reasons?

- To what extent the (COMPONENT) has answered your needs?
    - To what needs it answered in particular?
    - For what reasons did the (COMPONENT) answer or not your needs?

- Do you have the feeling that the (COMPONENT) has made a difference on your experience with the anti-hormonal drug?
    - For what reasons?
    - What positive/negative impacts of the (COMPONENT) have you perceived?

- Do you have any suggestions to improve (COMPONENT)? If yes, which ones?

- *For the education group session only:* During the education group session, a paper-based guide has been given to you. What do you think of this guide?
    - Have you consulted it up to now, at home? If yes, in which circumstances?
    - Was it helpful for you?
    - How could we improve it?

SATISFACTION REGARDING THE OTHER CARE AND SERVICES

- In addition to the SOIE program, what other care and services did you receive related to the anti-hormonal drug at the hospital or elsewhere?
    - Which care and services did you find the most useful? The less useful? For what reasons?

- Would you recommend adding the SOIE program to the usual care and services offered to women who have received an anti-hormonal drug?
    - If yes, what contribution of the program do you perceive compared to the other care and services?

CONCLUSION

- Do you have other ideas of how we could better support and equip women who receive an anti-hormonal drug following breast cancer?
- Is there anything you would like to share regarding your experience with the anti-hormonal drug, the care and services you have received related to the anti-hormonal drug, or the SOIE program?
