# Supplementary material for: Perspectives of Women with Breast Cancer and Healthcare Providers Participating in an Adherence-Enhancing Program for Adjuvant Endocrine Therapy: A Qualitative Study
Source: Curr Oncol. 2025 Jan 17;32(1):45. doi: 10.3390/curroncol32010045 (PMC11764025; doi:10.3390/curroncol32010045)
Supplement: Supplementary file 1 [file curroncol-32-00045-s001.zip › Supplemantary Materiel S2.pdf]

Supplementary Material S2

INDIVIDUAL INTERVIEW GUIDE
HEALTHCARE PROVIDERS

INTRODUCTION

- First, what role do you usually play with women with breast cancer? And what role do you have regarding the anti-hormonal drug?
- What role did you have in the delivery of the (COMPONENT)?

PREPARATION

- Have you participated in the training offered by the research team regarding the (COMPONENT)? And in the simulations of the (COMPONENT – education group session or chat sessions) before the delivery with the participants?
    - If yes, how did you find this training?
    - What do you think of the content/format/duration?
    - What did you think about its usefulness? Did you feel prepared enough to deliver the (COMPONENT) to the participants?
    - What facilitating/ constraining factors have you perceived regarding this training?
    - What are strengths of the training? And the points to improve?
    - Do you have any suggestions to better prepare healthcare professionals in delivering the (COMPONENT)? If yes, which ones?

- What do you think of the support offered by the research team for planning and delivering the (COMPONENT)?
    - What did you think about its usefulness? Did you feel supported enough to deliver the (COMPONENT) to the participants?
    - What facilitating/constraining factors have you perceived regarding this training?
    - What are strengths of the support? And the points to improve?
    - Do you have any suggestions to better support healthcare professionals in delivering the (COMPONENT)? If yes, which ones?

EXPERIENCE AND SATISFACTION WITH THE (COMPONENT)

- What did you think about the (COMPONENT)?
    - More specifically, about the format/times of the delivery/ duration/ number of participants/ frequency of the delivery/number of sessions or calls/delay between the sessions or calls/content presented to the participants/ material used for the delivery?

- Overall, how was the course of the (COMPONENT) that you delivered?
    - What receptiveness did you perceive from the women regarding the (COMPONENT)?
    - *For the education group sessions or chat sessions only:* How did the interactions between participants go?
    - *For the nurse-led telephone consultations only:* What do you think of the structure proposed for the calls? Of the use of the follow-up sheets?
    - *For the chat sessions only*: How did you find the use of the CancerChat Canada platform? How did the sharing of experience between participants go?

- How was your ease level in delivering the (COMPONENT)?

- What factors facilitated the delivery of the (COMPONENT)?

- Have you met difficulties in delivering the (COMPONENT)? If yes, which ones?
    - Have you tried strategies to face these difficulties? If yes, what results did you observe? If not, do you have any ideas on how these difficulties could be overcome?

- Have you collaborated with other healthcare providers in delivering the (COMPONENT)?
    - If yes, which ones and how?
    - How did you find your collaborative experience with this or these healthcare providers?
    - *For the education group sessions and chat sessions only:* How did the co-animation of the sessions go? What do you think of the number of animators/the disciplines of the healthcare providers chosen?
    - *For the nurse-led telephone consultations only:* How did the sharing of calls with you colleagues go?

- Do you have any suggestions for improvement? If yes, which ones?

IMPACTS

- Do you believe that the (COMPONENT) met the needs of participants?
    - What makes you believe this?
    - What could be done to make the (COMPONENT) better meet their needs?

- What positive/negative impacts of the (COMPONENT) have you perceived?
    - For the women/your professional practice/your healthcare team/the Breast Disease Center?

INTEGRATION OF THE (COMPONENT) AT THE BREAST CANCER DISEASE CENTER

- What do you think about the feasibility of integrating the (COMPONENT) into your usual practice? For what reasons?

- In your opinion, what factors could facilitate/hinder the integration of the (COMPONENT) into your usual practice/into the services offered? Do you have any suggestions to overcome the obstacles? If yes, which ones?

- What did you think of the workload related to (COMPONENT)?

- What did you think of the acceptability of the (COMPONENT) for patients at the Breast Disease Center?

OVERALL SATISFACTION OF THE (COMPONENT) AND CONCLUSION

- What do you think of the support and tools offered to women as part of the (COMPONENT)?

- Would you recommend the (COMPONENT) to women who have received a prescription for an anti-hormonal drug following breast cancer? For what reasons?

- Do any aspects of the SOIE program seem more/less useful to you? If yes, which ones? For what reasons?

- Is there anything else you would like to share about your experience with the (COMPONENT)?
